# Chemotherapy- and Immune-Related Gene Panel in Prognosis Prediction and Immune Microenvironment of SCLC

**DOI:** 10.3389/fcell.2022.893490

**Published:** 2022-06-15

**Authors:** Meng-Yu Chen, Yue-Can Zeng, Xi-He Zhao

**Affiliations:** ^1^ Department of Clinical Oncology, Shengjing Hospital of China Medical University, Shenyang, China; ^2^ Department of Breast Oncology, The Third Hospital of Nanchang, Nanchang, China; ^3^ Department of Radiation Oncology, Cancer Center, The Second Affiliated Hospital of Hainan Medical University, Haikou, China

**Keywords:** TOP2A, HLA, small-cell lung cancer, bioinformatics analysis, immune microenvironment

## Abstract

Small-cell lung cancer (SCLC) is a highly proliferative, invasive lung cancer with poor prognosis. Chemotherapy is still the standard first-line treatment for SCLC, but many patients relapse due to chemoresistance. Along with advances in immunology, it is essential to investigate potential indicators of the immune response and the prognosis of SCLC. Using bioinformatics analysis, we identified 313 differentially expressed genes (DEGs) in SCLC and normal lung samples, and we found that four upregulated genes (*TOP2A*, *CDKN2A*, *BIRC5*, and *MSH2*) were associated with platinum resistance, while immune-related genes (HLA family genes) were downregulated in SCLC. Then, a prognostic prediction model was constructed for SCLC based on those genes. Immune cell infiltration analysis showed that antigen presentation was weak in SCLC, and *TOP2A* expression was negatively correlated with CD8+ T cells, while *HLA-ABC* expression was positively correlated with M1 macrophages, memory B cells, and CD8+ T cells. We also found that *TOP2A* was related to poor prognosis and inversely correlated with *HLA-ABC*, which was verified with immunohistochemical staining in 151 SCLC specimens. Our study findings indicated that *TOP2A* may be a potential prognosis indicator and a target to reverse the immunosuppressive tumor microenvironment of SCLC.

## Introduction

SCLC is a lethal type of lung cancer with poor prognosis and represents approximately 15% of all lung cancers. It is highly aggressive and has a propensity to metastasize early (Gazdar et al., 2017; Blackhall et al., 2018). For more than 30 years, platinum (cisplatin or carboplatin) and etoposide chemotherapy has been administered as the standard first-line treatment for this disease; however, most cases are observed to relapse within 1 year of initial therapy due to chemoresistance (Waqar and Morgensztern, 2017). Recent advances in immunotherapy (e.g., PD-1, PD-L1, and CTLA-4 therapies) have shown promising results for patients with SCLC. Chemotherapy is combined with immunotherapy for SCLC treatment because the disease has a high tumor mutational burden; thus, chemotherapy can stimulate tumoral antigens and increase activation of T cells, thereby enhancing immunogenicity and priming the tumor for the response to immune checkpoint inhibitor treatment (Mak et al., 2019; Hiddinga et al., 2021). IMPOWER-133, a phase III trial, demonstrated that immunotherapy (atezolizumab or durvalumab) combined with platinum–etoposide chemotherapy achieved longer progression-free survival (PFS) (6.3 vs. 5.6 months) and patient overall survival (OS) (33.5 vs. 20.4% long-term survivors for control) compared with chemotherapy alone (Horn et al., 2018). Several other phase III studies (e.g., the CASPIAN trial and KEYNOTE-604 study) found that compared with treatment by chemotherapy alone, PFS and OS were significantly prolonged when immunotherapy and platinum-based front-line chemotherapy were combined (Paz-Ares et al., 2019; Rudin et al., 2020). However, results for maintenance immunotherapy after discontinuing first-line chemotherapy in the CheckMate-451 trial were unsatisfactory (Ready et al., 2016). Similarly, results for the CheckMate-331 trial showed that nivolumab as second-line therapy did not improve outcomes compared with the use of topotecan or amrubicin (Reck et al., 2018). Therefore, identifying biomarkers that could potentially identify patients who would benefit from immunotherapy is essential.

SCLC was previously classified into two subgroups, namely, classic and variant (Gazdar et al., 1985), and later, it was grouped into neuroendocrine and non-neuroendocrine categories (Zhang et al., 2018). At present, SCLC is grouped into four subtypes based on specific transcription factors: ASCL1 (SCLC-A), NEUROD1 (SCLC-N), POU2F3 (SCLC-P), and YAP1 (SCLC-Y) (Borromeo et al., 2016; Huang et al., 2018; Rudin et al., 2019). However, a newly proposed SCLC subtype “SCLC-I,” which is characterized by high expression of immune checkpoints or human leukocyte antigens (HLAs), has been noted to be correlated with cisplatin resistance and shows improved benefits from treatment using chemotherapy combined with immunotherapy (Gay et al., 2021). Of note, a subtype of SCLC has been noted to switch to another subtype under specific conditions, for example, cisplatin treatment of xenografts developed from patients with SCLC-A (MDA-SC68 model) enables SCLC-A to switch to SCLC-I, which suggests that targeting subtype transformations could be a means of regulating the mechanisms of the immune response and acquired platinum resistance (Gay et al., 2021). However, these classifications are controversial and require further investigation (Baine et al., 2020). A better understanding of antitumor immunity is also essential in order to elucidate the underlying mechanism of cancer immunosuppression and encourage biomarker development.

Topoisomerase II*α* (*TOP2A*) is a protein that is strongly expressed in proliferating cells, and it plays vital roles in regulating DNA replication, gene transcription, and mitosis (Tsai-Pflugfelder et al., 1988; Ali and Abd Hamid, 2016). Many studies have shown that *TOP2A* has the capacity to predict the sensitivity of breast cancer to anthracyclines. In addition, tumor cells with p53 mutations may exhibit high levels of *TOP2A* and may be more sensitive to *TOP2A* inhibitors (Liu et al., 2002); about 90% of patients with SCLC have p53 mutations (Delgado et al., 2005). High expression of *TOP2A* was reported to be associated with poor prognosis of NSCLC (Kou et al., 2020). However, only a few studies have investigated the clinical value of *TOP2A* expression in SCLC. Therefore, it is necessary to analyze the data of small-cell lung cancer obtained from existing databases to ascertain the clinical role of *TOP2A* in predicting survival outcome and immune response.

**FIGURE 1:**
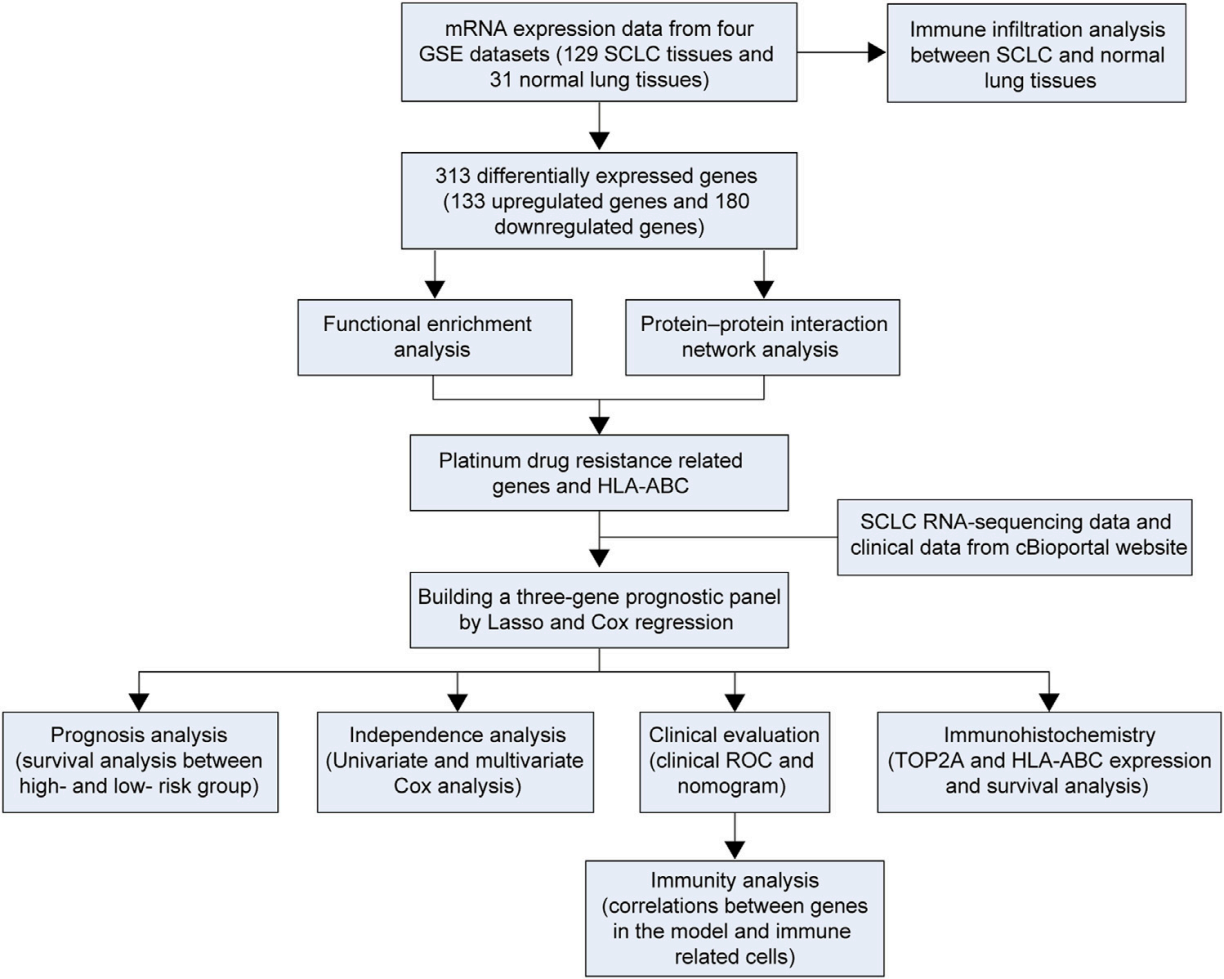
Flowchart of the study.

In this study, four GSE datasets [GSE6044 (Rohrbeck et al., 2008), GSE43346 (Sato et al., 2013), GSE60052 (Jiang et al., 2016), and GSE149507 (Cai et al., 2021)] were obtained from the GEO database to perform SCLC-related DEG analysis. A total number of 313 DEGs were identified in our study. Additionally, pathway enrichment and protein–protein interaction network (PPI) were conducted on the DEGs to select hub genes. Thereafter, a risk prediction score model was constructed. In addition, the clinical outcomes of different groups and correlations between genes in the model and immune-related cells were further investigated. Finally, immunohistochemistry analysis was performed to verify bioinformatics results. The study design is shown in Figure 1.

## Materials and Methods

### Small-Cell Lung Cancer Gene Expression Data Collection and Preprocessing

Four microarray expression profiles (i.e., GSE6044, GSE43346, GSE60052, and GSE149507) were obtained from the GEO database. The selected expression datasets satisfied the following criteria: 1) Studies were of human small-cell lung cancer tissues and corresponding para-cancerous tissues/normal lung tissues, 2) the number of samples included in each dataset was at least greater than 10, 3) the SCLC patients were naïve (untreated), and 4) the dataset search was limited to those studies written in English. The characteristics of the four selected datasets are shown in Table 1. For microarray data not shown in the form of log2-transformed values, log2 conversion was performed. If the data were not quantile-normalized, then the normalizeBetweenArrays method in the limma package of *R* was used for quantile normalization. The expression data were averaged for cases where multiple probes were mapped to one gene. Four GSE datasets were used to perform DEG identification, functional enrichment analysis, PPI network, and immune infiltration analysis in SCLC and normal lung tissue.

**TABLE 1:** Basic characteristics of four GSE datasets.

Dataset	Platform	Normal	Tumor	Reference
GSE6044	GPL201	5	9	Rohrbeck et al. (2008)
GSE43346	GPL570	1	23	Sato et al. (2013)
GSE60052	GPL11154	7	79	Jiang et al. (2016)
GSE149507	GPL23270	18	18	Cai et al. (2021)

### Identification of Small-Cell Lung Cancer Differentially Expressed Genes

DEGs were identified using the limma package (version 3.42.2) with the empirical Bayes method (Ritchie et al., 2015). Key parameters that selected DEGs conformed to the following criteria: |log2 fold change (FC)|>1 and adjusted *p* < 0.05. The RobustRankAggreg package (version 1.1) was used to integrate DEGs from four selected gene profiles. Heatmaps (package: pheatmap, version 1.0.12) and volcano plots (package: ggpubr, version 0.4.0) were used to visualize gene expression patterns. The common DEGs among these four datasets were obtained by using “Venn Diagram.”

### Functional Enrichment Analysis of Differentially Expressed Genes

A GO analysis including biological process (BP), cellular component (CC), and molecular function (MF) was performed to better explore potential biological function of DEGs. In addition, KEGG analyses were performed on the up- and downregulated genes using the Bioconductor package “clusterProfiler” (version 3.14.3). Notably, *q* value (adjusted *p* value) < 0.05 was used as a cutoff, and only GO terms and signal pathways passing this threshold were considered significant.

### Protein–Protein Interaction Network and Module Analysis

To better decipher the connections between the identified DEGs, the STRING biological database was utilized to construct the original PPI network. Only those DEGs with interaction scores >0.9 could be mapped into the network (Szklarczyk et al., 2019). Cytoscape 3.8.1 was utilized for the generation and visualization of the PPI network (Smoot et al., 2011). The top thirty genes with the maximum interactions were defined as hub genes using the cytoHubba plug-in of Cytoscape software (Chin et al., 2014). In addition, the highly interconnected clusters were extracted from the PPI network using another plug-in, MCODE.

### Construction and Verification of a Prognostic Model Based on Platinum-Related and HLA Class I Genes

We procured SCLC RNA sequencing as well as corresponding clinical data from the cBioPortal database for the construction of a prognostic model; FPKM values were subsequently transformed into TPM values. Samples were included when both mRNA-sequencing data and corresponding survival data of an SCLC patient were accessible (77 samples). For platinum-related genes (that were obtained from functional enrichment analysis) and HLA class I genes, we performed LASSO regression analysis (ten-fold cross confirmation and *p* < 0.05) using R package glmnet (version 4.1.1). Then, those selected genes were utilized to generate a prognostic model for SCLC using multivariate regression analysis (package: rms; version 6.2.0).

Next, we observed the survival differences between high- and low-risk subgroups (the median value of risk score was used as a cutoff) *via* Kaplan–Meier analysis by using the “survive” and “survminer” R packages. Next, the 1-, 3-, and 5-year receiver operating characteristic (ROC) curves of the proposed model for SCLC in comparison to other clinicopathological factors were drawn. Besides, univariate and multivariate Cox regression analyses were implemented to show whether our proposed model had predictive value for the prognosis of SCLC. A nomogram was generated to predict 1-, 3-, and 5-year OS of SCLC patients.

In addition, a chi-square test was conducted to disclose relationships between the model and other clinicopathological characteristics by using the “ComplexHeatmap” R package (version 2.2.0). Furthermore, a scatter diagram was used to visualize the Wilcoxon signed-rank test analysis results, which revealed differences in risk scores across distinct groups of clinicopathological characteristics.

### Survival Analysis of Genes Associated With Platinum Resistance and *HLA-B*

To show the independent prognostic potential of platinum resistance–related genes and genes in the proposed model (i.e., *MSH2*, *TOP2A*, *BIRC5*, *CDKN2A*, and *HLA-ABC*), we used survival and survminer packages to perform Kaplan–Meier analysis. Patients were clustered into two groups (high- or low-expression groups) based on the corresponding optimal cutoff value for each gene.

### Immune Infiltration Analysis

The immune infiltration analysis in SCLC and normal lung tissue was performed by using CIBERSORT (https://cibersort.stanford.edu), which could provide mRNA expression profiling of 22 immune cells. Notably, GSE6044 and GSE149507 only have 9 and 18 tumor samples, respectively. Given that analysis of few tumor samples may provide unreliable outcomes, we integrated four datasets to increase the total number of samples. The sva package (version 3.20) in R software (version 3.6.3; 64-bit) was applied to adjust potential batch effects. Besides, Spearman correlation analysis of the expression of the key genes (i.e., *TOP2A*, *CDKN2A*, *BIRC5*, *MSH2*, and *HLA-ABC*) and infiltrating immune cells was conducted by using the “ggpubr” package in the cBioPortal cohort.

### Immunohistochemistry

Paraffin-embedded specimens were obtained from 151 patients with primary SCLC confirmed by surgery or needle biopsy between April 2013 and October 2019 at the Department of Clinical Oncology, Shengjing Hospital of China Medical University. In addition, none of patients had secondary tumors or other severe diseases, nor did they receive preoperative chemotherapy or radiotherapy. Overall survival time of individuals was assessed from the surgery date to the date of event occurrence (death or last follow-up). The last follow-up was on 19 December 2021. All patients provided informed consent before surgery.

The specific steps for the immunohistochemistry (IHC) procedure were performed strictly according to the instructions. Paraffin-embedded sections underwent xylol dewaxing and rehydration routinely, and then endogenous catalase was inactivated with 3% H_2_O_2_ solution (15 min). For better exposing antigenic sites, the sections were immersed in a pre-heated citrate buffer (95–96°C, pH 6.1) for 20 min and then blocked in 5% normal goat serum to avoid unspecific binding (15 min). The sections were then incubated with anti-*TOP2A* antibodies (1:100, Proteintech, United States) or anti-*HLA-ABC* (1:5000, Proteintech, United States) antibodies at 4°C overnight and then incubated with biotin-labeled goat anti-mouse/rabbit IgG (Zhongshanjinqiao, Beijing, China) at 37°C for 120 min. DAB (Zhongshanjinqiao, Beijing, China) was used for detection.

Three random images of each SCLC section were taken at high-power fields (×200), and Image-Pro Plus (version 6.0) was used to measure the average optical density (IOD/area). Thus, the staining index of SCLC tissues was quantified. For *HLA-ABC* analysis, the assessment was primarily based on the presence of membranous staining, while *TOP2A* staining was mainly based on intracytoplasmic or intranuclear staining. A time-dependent ROC curve was used to better ascertain the cutoff value of *TOP2A* and *HLA-ABC* (package: survivalROC, version 1.0.3).

### Statistical Analysis

The χ^2^ test was utilized to disclose associations between *TOP2A*, *HLA-ABC*, and categorical variables (SPSS, Chicago, IL, United States, version 3.6.3). Survival data of patients with SCLC were analyzed using the Kaplan–Meier method, and comparison of different survival times between groups was drawn using the log-rank test (package: survminer, version 0.4.8). Correlation analysis between *TOP2A* and *HLA* was conducted using the Pearson method (SPSS) in datasets obtained from GSE datasets and the cBioPortal website, while Spearman rank correlation was used in our own cohort (two-sided *p* values and *p* < 0.05 was considered statistically significant).

## Results

### Identification of Differentially Expressed Genes

**FIGURE 2:**
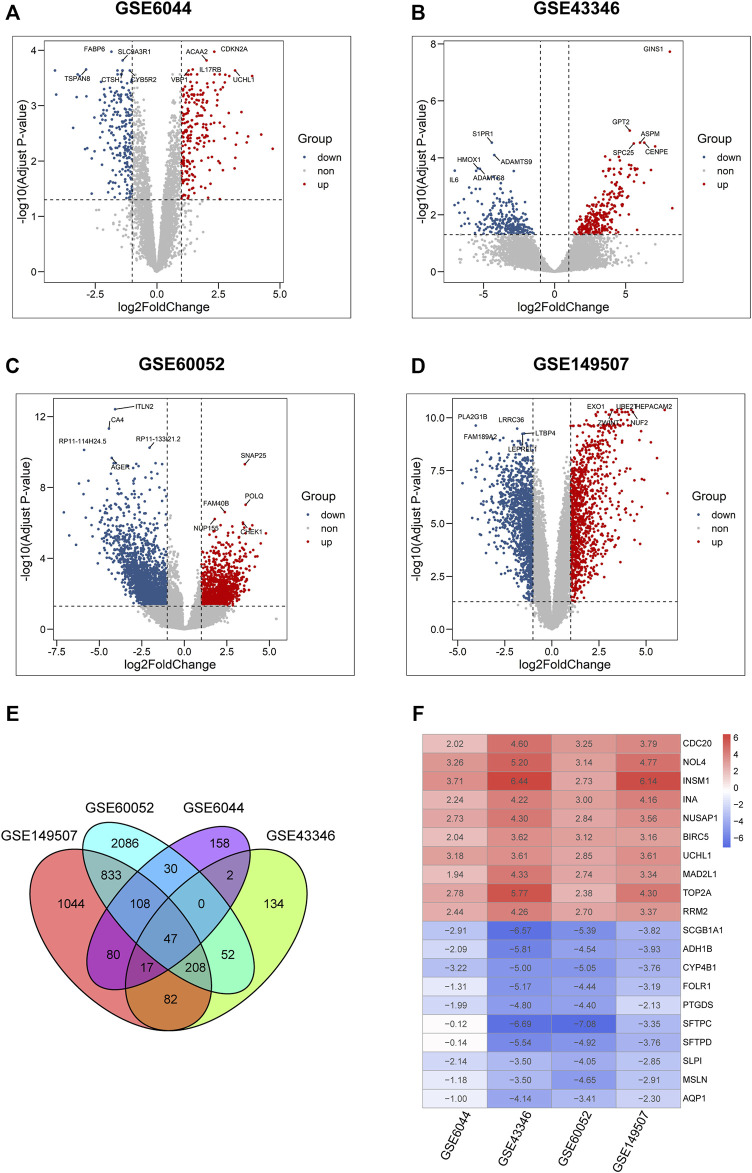
Identification of DEGs in four microarray datasets from GEO. **(A–D)** Volcano plots of differential expression analysis for GSE6044, GSE43346, GSE60052, and GSE149507. Plots in red, blue, and gray represent upregulated, downregulated, and non-significant genes, respectively. **(E)** Venn diagram of the overlapped differential expressed genes in four GSE datasets. **(F)** Expression heatmap of top 10 up- and down-regulated genes, each column in the table represents a Log2FC value calculated for each gene.

Four microarray datasets (GSE6044, GSE43346, GSE60052, and GSE149507) were used to analyze DEGs, and volcano plots of DEGs for each dataset are presented in Figures 2A–D. The overlap of these DEGs was examined using a Venn diagram (Figure 2E). Then, these DEGs were integrated using the robust rank aggregation algorithm of the RobustRankAggreg package to obtain 313 DEGs (tumor vs. normal; 133 upregulated DEGs and 180 downregulated DEGs, respectively). A heatmap of the DEGs is shown in Figure 2F. The top 10 upregulated genes were *CDC20*, *NOL4*, *INSM1*, *INA*, *NUSAP1*, *BIRC5*, *UCHL1*, *MAD2L1*, *TOP2A*, and *RRM2*, while the top 10 downregulated genes were *AQP1*, *MSLN*, *SLP1*, *SFTPD*, *SFTPC*, *PTGDS*, *FOLR1*, *CYP4B1*, *ADH1B*, and *SCGB1A1*.

### Function and Pathway Enrichment Analyses of Differentially Expressed Genes

**FIGURE 3:**
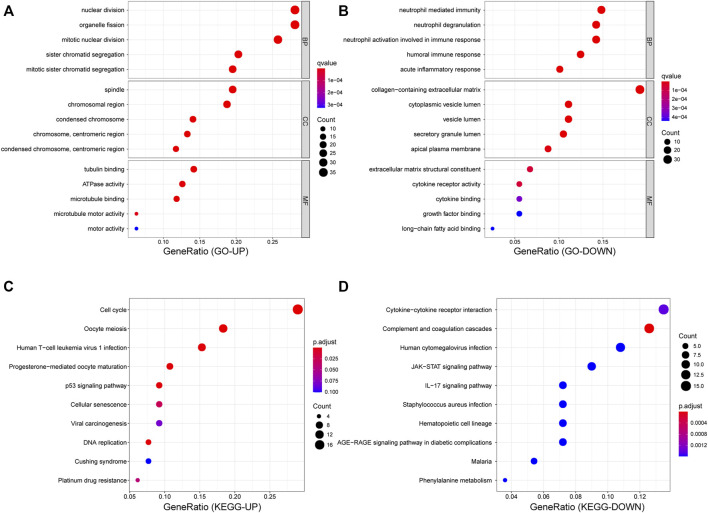
Functional enrichment analyses for DEGs. **(A,B)** Dot plot of GO enrichment analysis of up- and down- regulated genes. **(C,D)** Dot plot of KEGG enrichment analysis of up- and down-regulated genes.

To shed light on the biological roles of DEGs in patients with SCLC, we performed GO and KEGG enrichment analyses for both upregulated and downregulated genes. Results of the GO analysis revealed that upregulated DEGs mainly focused on the cell mitosis process and microtubule motor activity (Figure 3A), and the KEGG analysis revealed that those genes were primarily mapped to the process related to cell cycle, DNA replication, p53 pathway, and platinum drug resistance (Figure 3C). In contrast, among the downregulated DEGs, GO terms showed significant enrichment in neutrophil-mediated immunity (Figure 3B), while KEGG analysis also showed enrichment in cytokine–cytokine receptor interaction and activation of transcription and the interleukin-17 signaling pathway (Figure 3D).

### Protein–Protein Interaction Network and Hub Genes

**FIGURE 4:**
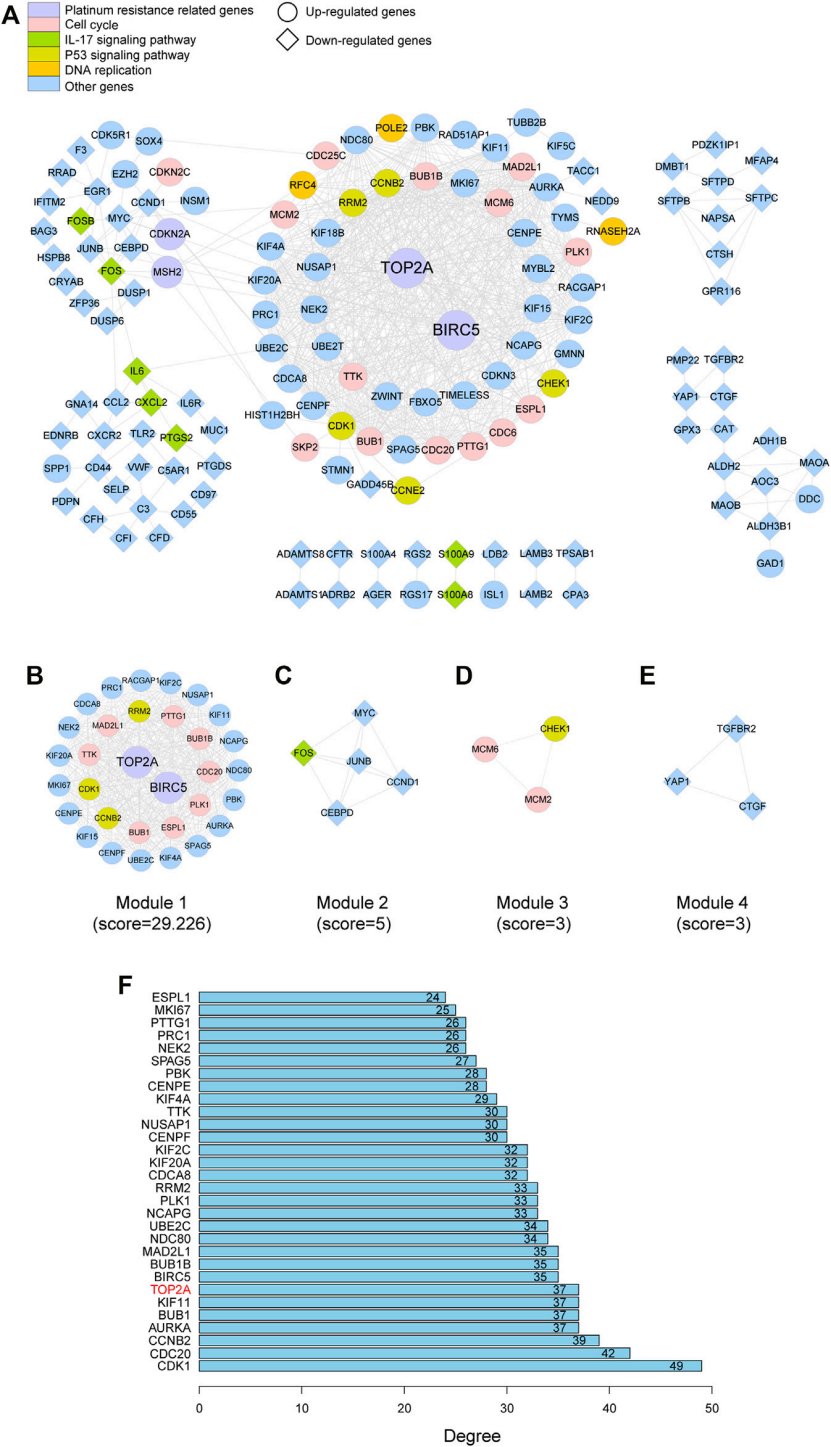
PPI network of the DEGs and hub gene identification. **(A)** Visualization of the PPI network of DEGs identified in samples from GSE datasets. **(B–E)** Network of four functional clusters that were identified by MCODE. **(F)** Top 30 genes with high degree calculated by cytoHubba.

As depicted in Figure 4. 313 nodes and 1,548 edges were involved in the PPI network. The top 30 hub genes were screened on the basis of connectivity degree in this network (Figure 4F). The MCODE plug-in was then optimized to identify clusters in the network; four clusters were identified using k-core = 2 (Figures 4B–E). Cluster 1 had 32 nodes and 453 edges; it was the highest scoring cluster among those identified (Figure 4B). Core genes were also screened based on functional enrichment analysis and candidate hub genes. Our KEGG analysis further investigated four platinum resistance–related genes, that is, *TOP2A*, *CDKN2A*, *BIRC5*, and *MSH2*. Among these, *TOP2A* and *BIRC5* were included in module 1 and with 37 and 35 connections, respectively, suggesting that they potentially play critical roles in SCLC. Notably, *HLA-DMA* was downregulated in SCLC tissues in contrast with its expression in normal tissues (logFC = −1.7849; *p* = 0.018). HLA genes are known to have an important function in immune response; therefore, to further investigate the expression of HLA family genes in SCLC, we searched each GEO dataset to find neglected information. We found that *HLA-E* was downregulated in GSE6044; *HLA-E*, *HLA-DQB1*, *HLA-DPB1*, and *HLA-DMA* were downregulated in GSE149507; and *HLA-B*, *HLA-DQB1*, *HLA-DQB2*, *HLA-E*, *HLA-DMA*, *HLA-DPA1*, *HLA-DPB1*, *HLA-DRA*, *HLA-DRB1*, *HLA-DRB6*, and *HLA-DOA* were downregulated in GSE60052; in contrast, HLA-related DEGs were not found in GSE43346. In summary, *TOP2A* and *BIRC5* were upregulated in SCLC, whereas HLA family genes were possibly downregulated in SCLC.

### Construction and Verification of a Prognostic Panel by Chemotherapy-Related Genes and HLA-I Genes

**FIGURE 5:**
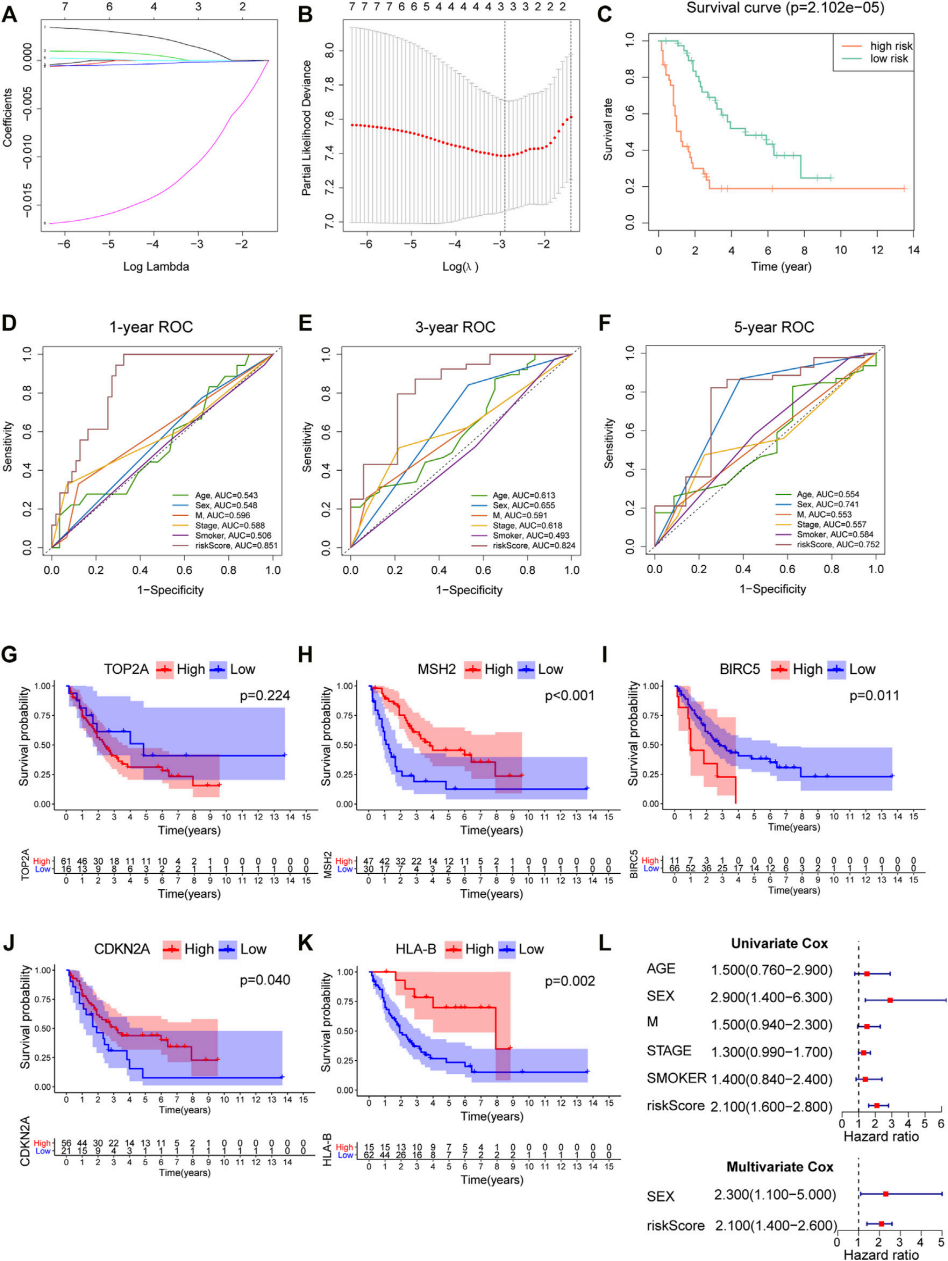
Construction and verification of a prognostic model for SCLC. **(A)** Coefficient patterns of the candidate 7 variables in the LASSO model. **(B)** Tenfold cross validation was applied to select the proximal turning parameter (*λ*) *via* minimum criteria (the 1-SE criteria) in the LASSO model. **(C)** Kaplan–Meier curves of 77 SCLC patients stratified by the median value of risk score. **(D–F)** 1-, 3-, and 5-year ROC curves of risk score, age, sex, distant metastasis, clinical stage, and smoking history in the cBioPortal cohort. **(G–K)** Survival analysis of platinum resistance–related genes and HLA-B in 77 SCLC patients; the median expression of each gene was used as the cutoff. **(L)** Forest maps of univariate and multivariate Cox regression in the cBioPortal cohort.

We screened out five genes (i.e., *TOP2A*, *BIRC5*, *CDKN2A*, *MSH2*, and *HLA*-ABC) for further LASSO regression in light of the above findings. According to the optimal value of *λ*, the three-gene–based signature had superior predictive value (Figure 5B), and coefficients for each gene are presented in Figure 5A. Finally, a prognostic prediction panel was established. For each patient, the risk score was computed as follows:

$$\begin{array}{l} \mathrm{Risk}\;\mathrm{score}=\mathrm{TPM}\;\mathrm{value}\;\mathrm{of}\;\mathrm{TOP}2A\times 0.002566-\mathrm{TPM}\;\mathrm{value}\;\mathrm{of}\;\mathrm{HLA}\\ -B\times 0.00022-\mathrm{TPM}\;\mathrm{value}\;\mathrm{of}\;\mathrm{MSH}2\;\times \;0.0154 \end{array}$$

According to the median levels of risk score, patients were dichotomized into high- and low-risk groups. As shown in Figure 5C, patients with higher risk had significantly worse survival. Besides, our model outperformed other variables including patient age, gender, smoking, and metastasis status (Figures 5D–F), with an AUC = 0.851, 0.824, and 0.752 for 1, 3, and 5 years, respectively. In addition, survival analysis was employed to analyze platinum resistance–related genes (i.e., *HLA-B*, *TOP2A*, *MSH2*, *BIRC5*, and *CDKN2A*) and prognosis (Figures 5G–K). Patients with high expression of *HLA-B*, *MSH2*, and *CDKN2A* expression had better survival. Although *p* value for *TOP2A* was greater than 0.05, it can be seen from Figure 5G that the long-term survival of patients with high expression of *TOP2A* was worse than those with lower expression. Furthermore, results showed that a high risk score denoted worse prognosis, and this proposed model was predictive of outcomes in SCLC (Figure 5L).

### Clinical Evaluation of the Model

**FIGURE 6:**
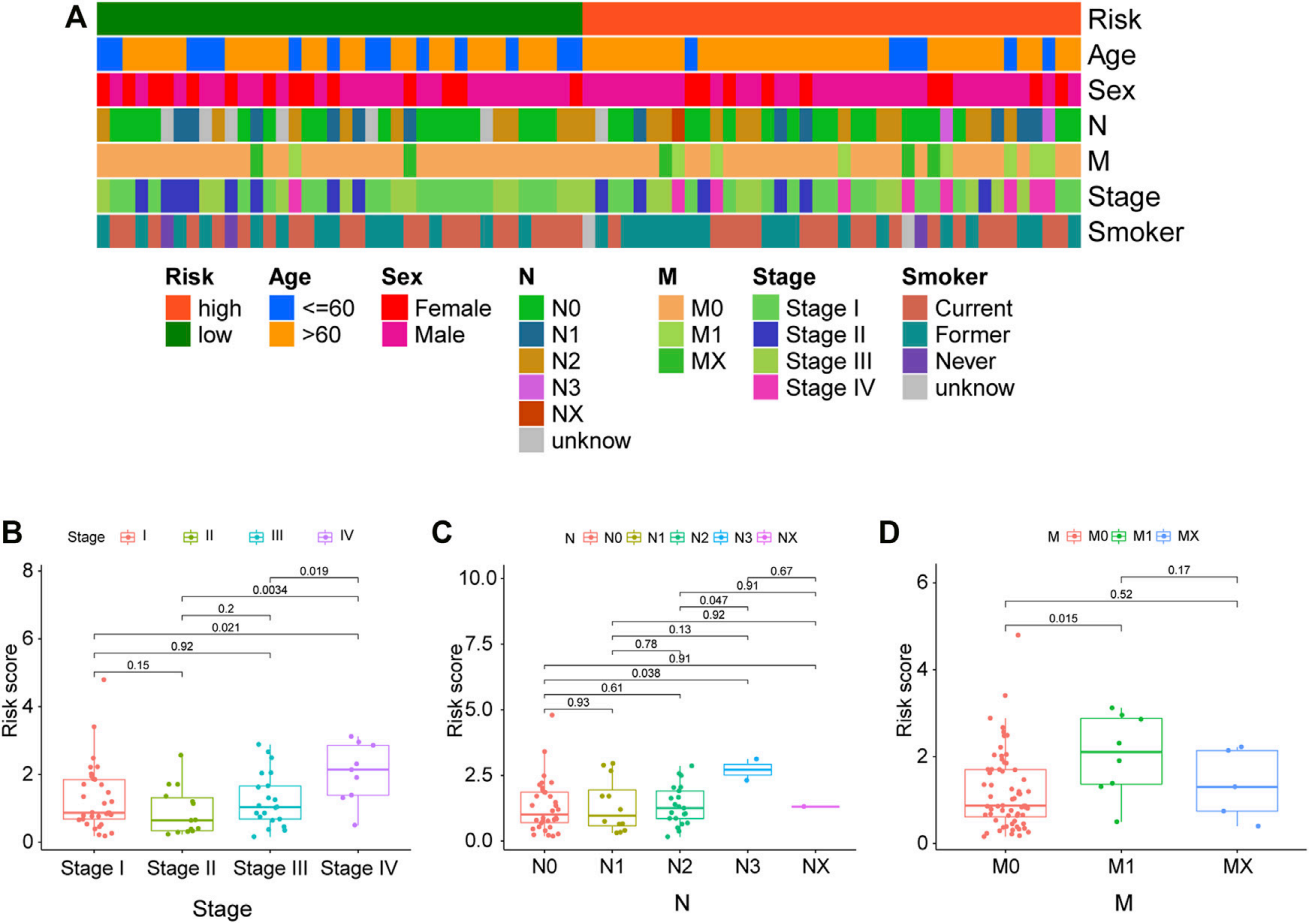
Correlations between clinical characteristics and risk score in the cBioPortal cohort. **(A)** The correlations between various clinical characteristics and risk score. Correlation coefficient and *p* values were determined by the Spearman rank correlation test. *p* < 0.001 = ∗∗∗, *p* < 0.01 = ∗∗, and *p* < 0.05 = ∗. **(B–D)** Correlations between the risk score and different tumor stages, N stage, and M stage.

**FIGURE 7:**
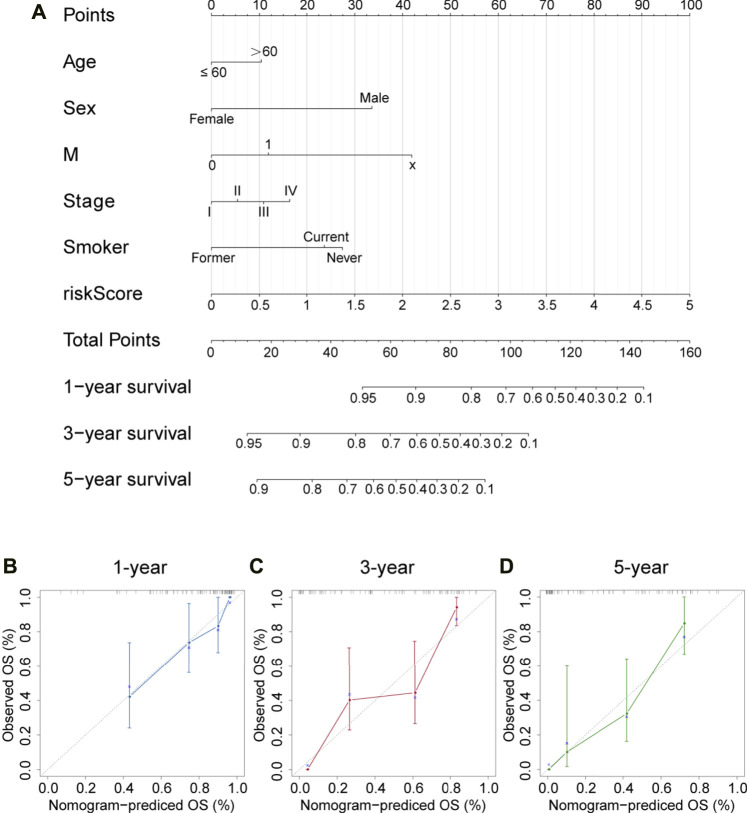
Nomogram and corresponding calibration plots. **(A)** A nomogram that integrated the risk score and other clinicopathological features for predicting 1-, 3-, and 5-year OS of SCLC. Scores of each variable are calculated by drawing a line vertical to the top points row, and then a total score can be obtained by summing up these scores. Subsequently, a vertical line could be projected from total points to the bottom scales to estimate the survival probability of patients. **(B–D)** Calibration plots for 1-, 3-, and 5-year nomogram in the cBioPortal cohort. Nomogram-predicted survival is depicted on the *X*-axis, and observed actual survival is plotted on the *Y*-axis. The 45-degree dotted line indicates a perfect prediction.

Correlations between the model and various clinicopathological characteristics were further investigated by using the chi-square test; however, results indicated that the risk score was not significantly associated with clinicopathological factors (Figure 6A). Then, the Wilcoxon signed-rank test was performed to analyze possible discrepancy in risk score among subgroups stratified by clinicopathological characteristics. As indicated in Figures 6B–D, stage IV had a higher risk score than stage I–III. Similarity, N0–2 also showed no differences, but they had a much lower risk score than N3. Besides, distant metastasis was positively correlated with risk scores. A nomogram based on the panel and other traditional clinicopathological characteristics was constructed, as shown in Figure 7A. The calibration curve showed adequate fit of the proposed nomogram model in predicting 1-, 3-, and 5-year OS of SCLC (Figures 7B–D).

### Immune Infiltration Analysis

**FIGURE 8:**
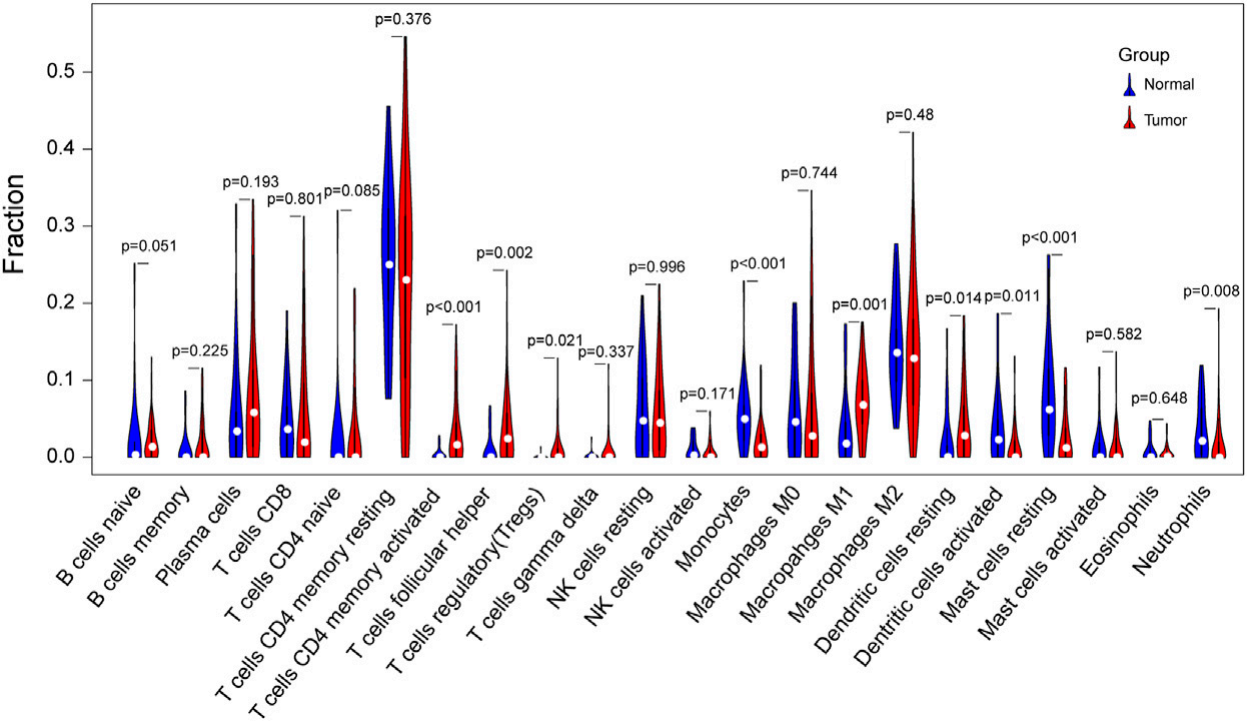
Violin plot of differences in immune cell infiltration between SCLC and normal lung tissues. Comparison of 22 infiltrated immune cells in tumor samples and normal lung tissues based on four GSE datasets. Blue color represents normal lung tissue, while red represents tumor tissue.

Given that the downregulated DEGs showed enrichment in immune-related pathways, immune infiltration analysis was performed in SCLC and normal samples. Interestingly, we found that SCLC tissues had a higher proportion of activated memory CD4^+^ T cells, T-follicular helper cells, regulatory T cells, M1 macrophages, and resting dendritic cells than that detected in normal lung tissues. In contrast, the proportion of monocytes, activated dendritic cells, resting mast cells, and neutrophils was much lower in SCLC tissues (Figure 8).

**FIGURE 9:**
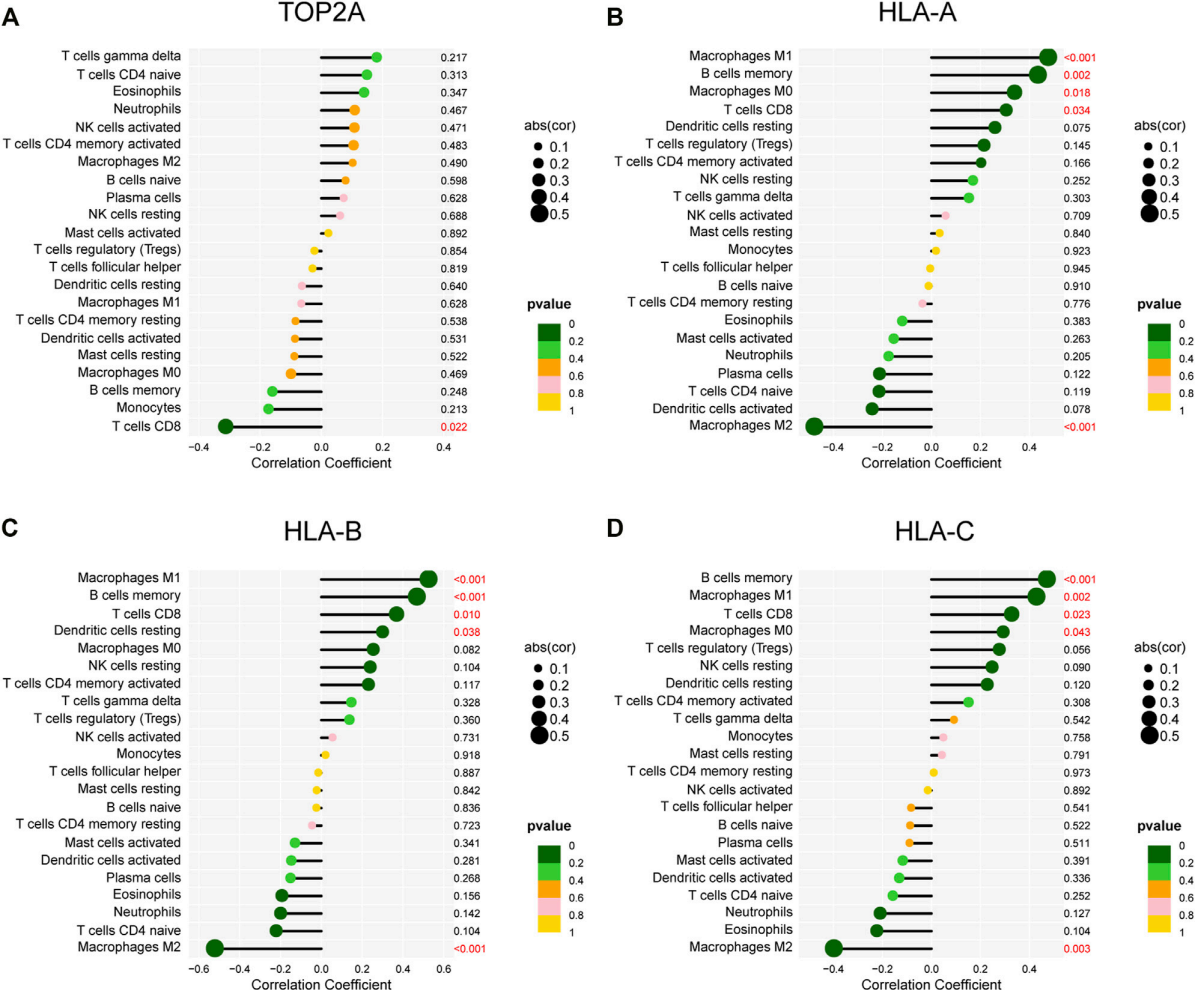
Correlations of gene expression and infiltrating immune cells. **(A–D)** Correlations between *TOP2A*, *HLA-A*, *HLA-B*, and *HLA-C* expression and infiltrating immune cells.

A lollipop plot was drawn to show correlations between expression of those key genes (i.e., *TOP2A*, *CDKN2A*, *BIRC5*, *MSH2*, and *HLA-ABC*) and immune-related cells. As shown in Figure 9A, *TOP2A* expression was negatively correlated with CD8+ T cells; *HLA-A*, *HLA-B*, and *HLA-C* expression had significant positive correlations with M1 macrophages, memory B cells, and CD8+ T cells. Besides, they all were inversely associated with M2 macrophages (Figures 9B–D). In addition, both *HLA-A* and *HLA-C* expression were positively correlated with M0 macrophages, while *HLA-B* expression was positively correlated with resting dendritic cells. However, *CDKN2A*, *BIRC5*, and *MSH2* expression showed no correlations with immune cells (Supplementary Tables S1–S3).

### Correlations Between *TOP2A* and *HLA-I* Expression in Small-Cell Lung Cancer

To investigate the association between *TOP2A* and *HLA-ABC*, Pearson correlation analysis was applied in GEO datasets and the cBioPortal cohort. Of note, the correlation coefficients of *TOP2A* with *HLA-ABC* were all negative (Table 2). However, statistically significant results were only found in GSE60052, which may have happened because of the large sample size of this dataset (Table 2). Notably, in our cohort, *TOP2A* expression was significantly inversely correlated with HLA (Table 3).

**TABLE 2:** Correlation between *TOP2A* expression and *HLA-ABC* expression in four GSE datasets and the cBioPortal website.

Dataset (*TOP2A*)	*HLA-A*		*HLA-B*		*HLA-C*	
	*R*	*p*	*R*	*p*	*R*	*p*
GSE6044	−0.55	0.12	−0.57	0.11	−0.36	0.33
GSE43346	−0.32	0.14	−0.12	0.34	−0.36	0.087
GSE60052	−0.22	**0.049**	−0.19	0.086	−0.19	0.096
GSE149507	−0.31	0.21	NA	NA	−0.3	0.22
cBioPortal	−0.22	0.055	−0.127	0.273	−0.209	0.068

Bold font indicates statistically significant p value (<0.05).

**TABLE 3:** Correlation between *TOP2A* expression and *HLA-ABC* expression in our own cohort.

	*TOP2A* (high)	*TOP2A* (low)	Total
*HLA-ABC* (high)	20 (13.2)	29 (19.2)	49 (32.5)
*HLA-ABC* (low)	62 (41.1)	40 (26.5)	102 (67.5)
Total	82 (54.3)	69 (45.7)	151

*R* = −0.188. *p* = 0.021. Data are expressed as *n* (%).

### Survival Analysis of *TOP2A* and *HLA-ABC* Expression for Small-Cell Lung Cancer Patients

**FIGURE 10:**
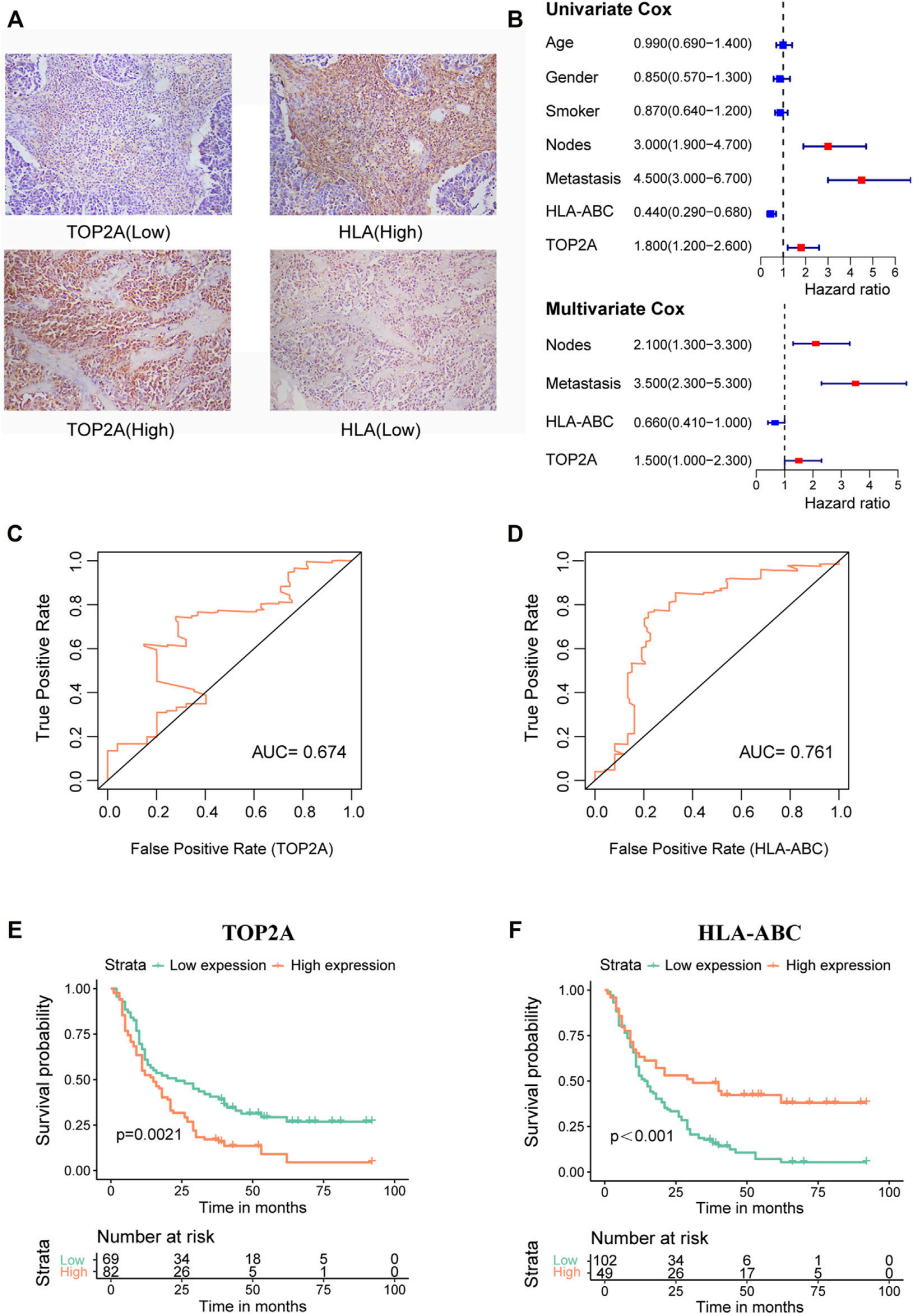
Verification of *TOP2A* and *HLA-ABC* expression by immunohistochemistry in our SCLC cohort. **(A)** Representative immunohistochemical pictures of *TOP2A* and *HLA-ABC* in SCLC tissue sections. **(B)** Forest plot for univariate and multivariate Cox regression analysis. **(C,D)** Time-dependent receiver operating characteristic curves of *TOP2A* and *HLA-ABC*. **(E,F)** Kaplan–Meier survival curve for 151 SCLC patients stratified by the cutoff values of *TOP2A* and *HLA-ABC*, respectively.

The patient group (*n* = 151) comprised 105 males and 46 females, ranging in age from 37 to 79 years (mean ± SD, 61.27 ± 8.323 years old). Additionally, there were 109 patients (72.19%) who had lymph node metastases (LNM) and 104 patients (68.87%) had distant metastasis. *TOP2A* and *HLA-ABC* expression levels were analyzed semiquantitatively using the average optical density viewed in Image-Pro Plus after immunohistochemical staining. Representative pictures of IHC staining of SCLC are shown in Figure 10A, and more immunohistochemical pictures are shown in Supplementary Figure S1. Time-dependent ROC curves showed that the best critical cutoff values for *TOP2A* and *HLA-ABC* were 0.00498 and 0.00731, respectively (Figures 10C,D). The reciprocal of *HLA-ABC* (1/*HLA-ABC*) was used to improve the comparability of the ROC curves because this gene was downregulated in SCLC and may therefore be a protective factor. Thus, according to expression levels of *TOP2A* and *HLA-ABC*, patients were classified into high- or low-expression groups. Fifty-four percent of the patients had high *TOP2A* expression levels, while 46% of patients had low levels of *TOP2A*; in contrast, high and low expression of *HLA-ABC* was found in 32.5 and 67.5% patients, respectively. Correlations between *TOP2A*/*HLA-ABC* expression and clinicopathological characteristics of 151 patients with SCLC are shown in Table 4. However, there was no appreciable difference in age, sex, history of smoking, or lymph node metastasis between *TOP2A*/*HLA-ABC* high- and low-expression groups (*p* > 0.05).

**TABLE 4:** Correlations between *TOP2A/HLA-ABC* expression and clinicopathological characteristics of 151 patients with SCLC.

	N	*TOP2A*				*HLA-ABC*			
		Low (N = 69)	High (N = 82)	X^2^	*p*	Low (N = 102)	High (N = 49)	X^2^	*p*
Gender									
Male	105	44	61	1.996	0.158	70	35	0.726	0.123
Female	46	25	21	—	—	32	14	—	—
Age									
≤60	63	27	36	0.351	0.554	45	18	0.742	0.389
>60	88	42	46	—	—	57	31	—	—
Smoker									
Yes	79	37	42	*	0.307	51	28	*	0.482
No	65	27	38	—	—	45	20		
Ever	7	5	2	—	—	6	1		
N stage									
N0	42	21	21	0.434	0.510	24	18	2.875	0.09
N1–3	109	48	61	—	—	78	31		
M stage									
M0	104	48	56	0.028	0.866	67	37	1.49	0.222
M1	47	21	26	—	—	35	12		

Note: “*” means it is calculated using Fisher’s exact test.

Additionally, low expression of *TOP2A* and high expression of *HLA-ABC* predicted longer OS as indicated in Figures 10E,F. Univariate Cox analysis showed that LNM (HR = 3.000; *p* < 0.05), distant metastasis (HR = 4.500; *p* < 0.05), and high expression of *TOP2A* (HR = 1.800; *p* < 0.05) portended a poor prognosis, while high expression of *HLA-ABC* denoted a favorable prognosis. Multivariate Cox analysis showed that the risk of death was 2.1 times higher in patients with LNM than those without LNM and 3.5 times higher for patients with M1 stage than those with M0 stage. Patients with high expression of *TOP2A* had 1.5-fold higher mortality risk than patients with low expression, while patients with high *HLA-ABC* expression harbored a 0.66-fold lower risk of death. Therefore, *TOP2A*, *HLA-ABC*, lymph node metastasis, and distance can be regarded as independent prognostic factors of SCLC (Figure 10B).

## Discussion

Tumor typing of solid tumors (from the initial location distinction to the pathological type distinction as well as the recent molecular classification distinction) is known to be an effective method for cancer treatment. SCLC is a malignant type of cancer with obvious genetic characteristics, but targeting SCLC treatment remains challenging. Notably, the neuroendocrine types of SCLC (i.e., SCLC-A and SCLC-N) can be regarded as cold tumors, which are characterized by the infiltration of immune cells into the tumor center and the invasive margin; in contrast, non-neuroendocrine types (i.e., SCLC-Y, SCLC-P, and SCLC-I) manifest a phenotype that is more inflamed (Galon and Bruni, 2019; Haryono et al., 2019; Gay et al., 2021). Transformability between different subtypes may provide new treatment opportunities for patients with SCLC who respond poorly to immunotherapy (Lim et al., 2017; Ireland et al., 2020). Therefore, bioinformatics analysis was performed in this study to investigate underlying predictors of immunotherapy in SCLC. Data were downloaded from the GEO database and the cBioPortal website.

We identified 133 and 180 upregulated and downregulated DEGs, respectively, that were potentially associated with the development of SCLC. Notably, four upregulated DEGs were enriched in the platinum drug resistance pathway. In addition, human leukocyte antigen class I and II (*HLA-I* and *HLA-II*) were downregulated in SCLC tissues compared with normal lung tissues. According to enrichment analysis, the DEGs were enriched in DNA replication, mitotic nuclear division, and chromosome segregation, whereas downregulated genes were associated with neutrophil-mediated immune response. CIBERSOET analysis was used to delineate the immune infiltration landscape in SCLC tissues. SCLC seemed to have a pauci-immune microenvironment that was consistent with GO and KEGG analysis results. The lower proportion of monocytes, activated dendritic cells, and neutrophils in SCLC compared with that in normal lung tissues implied weak antigen-presenting capacity in SCLC. These results suggest that although SCLC had a high mutational burden, the efficacy of immunotherapy was not always sufficient even when it was combined with chemotherapy, and it is greatly affected by the interplay of tumor cells and the immune system in the tumor microenvironment. Thus, we used LASSO and multivariate Cox regression to construct a prognostic index based on four chemotherapy-related genes and *HLA-ABC* genes. Results showed that this model was based on the TPM value of three genes: *HLA-B*, *MSH2*, and *TOP2A*. Notably, the coefficients were positive for *HLA-B* and *MSH2*, whereas they were negative for *TOP2A*. Patients with higher risk scores had less favorable outcomes.

*TOP2A* expression induces the formation of covalent complexes with DNA, and produces transient double-stranded DNA breaks (DSBs), which are crucial for DNA metabolism processes including replication, chromosome condensation, and chromatid separation during mitosis (Deweese and Osheroff, 2009; Jain et al., 2013). Previous studies have suggested that *TOP2A* was upregulated and indicative of poor prognosis in many malignancies including lung adenocarcinoma (Kou et al., 2020), gastric cancer (Cao et al., 2017), breast cancer (Zheng et al., 2016), and prostate cancer (de Resende et al., 2013), which is noteworthy. Generally, patients with SCLC respond well to initial chemotherapy but show poor prognosis. Patients with SCLC who experience a clinical relapse (i.e., patients who are drug resistant) rarely achieve an objective response rate greater than 20% in their second-line treatment (von Pawel, 2003). Indeed, combination treatment with immunotherapy is often ineffective, especially after clinical trials of anti-PD-1 antibodies in SCLC ended with failure. This seemingly contradictory situation may actually stem from the inherent relationship between *TOP2A* and *HLA* genes. When *TOP2A* is upregulated, *HLA* expression is usually downregulated, and this was confirmed by immune infiltration results that *TOP2A* expression was negatively correlated with CD8+ T cells. Weakened antigen presentation leads to immune evasion and metastasis of tumor cells, which in turn leads to multi-therapeutic resistance. Inhibition of *TOP2A* expression alone or combined with immunotherapy may be promising treatment options for SCLC.

*MSH2* is one of the DNA mismatch repair (MMR) genes, which is upregulated in many cancers including SCLC (Fujii et al., 2018). *MSH2* mediates the removal of platinum–DNA adducts. However, platinum agents induce apoptosis by generating covalent platinum–DNA adducts that block DNA replication and transcription (Barry et al., 1990). Emerging evidence demonstrates that patients with *MSH2* loss had a low response rate to platinum-based therapies in many malignancies, including glioma and ovarian cancer (Pabla et al., 2011; Goodspeed et al., 2019). However, more studies are needed to explore whether *MSH2* can be implicated in resistance to chemotherapy in SCLC.

*HLA-I* is composed of three classical antigens (*HLA-A*, *-B*, and *-C*); it is present on the cell surface of every human cell and enables peptides derived from tumor cells to be recognized by cytotoxic T-lymphocytes (CTLs) while also playing a critical role in antitumor immunity. Cancer cells downregulate *HLA-I* expression by destroying the stability of *β*2-microglobulin, which could cause the loss of heterozygosity (LOH) of *HLA-I* (Challa-Malladi et al., 2011; Bernal et al., 2012)*.* Loss of *HLA-I* expression often occurs in many malignancies, including SCLC; it results in resistance to the activity of *HLA*-restricted CTLs, which leads to T-cell–mediated immune evasion and dissemination of tumor cells (Doyle et al., 1985; Garrido et al., 2016). Chowell et al. (Chowell et al., 2018) found that LOH of *HLA-I* in patients was predictive of poor survival in comparison with patients without such LOH. Moreover, Rodig et al. (Rodig et al., 2018) demonstrated that loss of *HLA* expression might influence immune checkpoint blockade responses; specifically, the initial response to anti-CTLA-4 required MHC-I–mediated antigen presentation, whereas intact expression of tumor-specific MHC-II molecules was needed for the anti-PD-1 response in melanoma. Thus, *HLA* expression was positively associated with immune therapy effects.

Based on functions of *TOP2A* in malignant neoplasm progression and platinum resistance, and *HLA-ABC* expression in the immune response, the correlation between *TOP2A* and *HLA-I* was investigated. Intriguingly, we found negative correlations between *TOP2A* and *HLA-I*, and this was confirmed by our immunohistochemistry experiments*.* Furthermore, survival analysis based on SCLC data from the cBioPortal website showed that *HLA-B* was an independent indicator of good prognosis, while *TOP2A* might be associated with poor long-term survival of patients. Interestingly, our study indicated that low and high expression levels of *TOP2A* and *HLA*-*ABC*, respectively, were significantly associated with increased OS in patients with SCLC. Downregulation of *TOP2A* may have the potential to turn the *TOP2A*-positive/*HLA-I*–negative phenotype into the *HLA*-*ABC*–positive/*TOP2A*-negative phenotype, which could promote the tumor immune response. In *TOP2A*-positive/*HLA-I*–negative cases, patients with SCLC may have poor prognosis; in contrast, in *TOP2A*-negative/*HLA-I*–positive cases, patients may achieve increased survival and benefit from immunotherapy, which is in accordance with coefficients of the corresponding gene in our proposed model. Moreover, mutual transformations could occur among the various subtypes found in patients with SCLC. Gene therapy for *TOP2A* and *HLA-ABC* may enable patients with recurrence and metastasis to become treatment-sensitive and thereby benefit from chemotherapy with or without immunotherapy. Moreover, specific genomic characteristics of SCLC and corresponding individualized treatments should be focused upon in order to ultimately improve the current therapeutic landscape.

There are still some limitations to this study. First, we used only one antibody that recognizes the non-polymorphic region of *HLA-I*, which means that different HLA loci might be associated with different clinical characteristics. Second, our sample numbers are small due to the rarity of samples; more multicenter and larger-scale studies are required to investigate findings and the hypothesis in the current study.

## Conclusion

Our results created a risk model based on chemotherapy-related genes and immune-related genes for SCLC patients; this model may be beneficial for evaluating SCLC patient prognosis. In addition, *TOP2A* was inversely correlated with *HLA-ABC* in SCLC, which indicated that *TOP2A* may be a potential predictive factor of immune response. The upregulation of *TOP2A* together with loss of *HLA-ABC* in SCLC are associated with poor prognosis, and both of them are unfavorable independent prognosticators for SCLC. Our study offers a potential target to reverse the immunosuppressive tumor microenvironment of SCLC, and provides new insight into overcoming the predicament of SCLC clinical therapy.

## Data Availability

The datasets presented in this study can be found in online repositories. The names of the repository/repositories and accession number(s) can be found in the article/Supplementary Material.
