# Supplementary figures and images for: Chemotherapy- and Immune-Related Gene Panel in Prognosis Prediction and Immune Microenvironment of SCLC
Source: Front Cell Dev Biol. 2022 Jun 15;10:893490. doi: 10.3389/fcell.2022.893490 (PMC9240612; doi:10.3389/fcell.2022.893490)

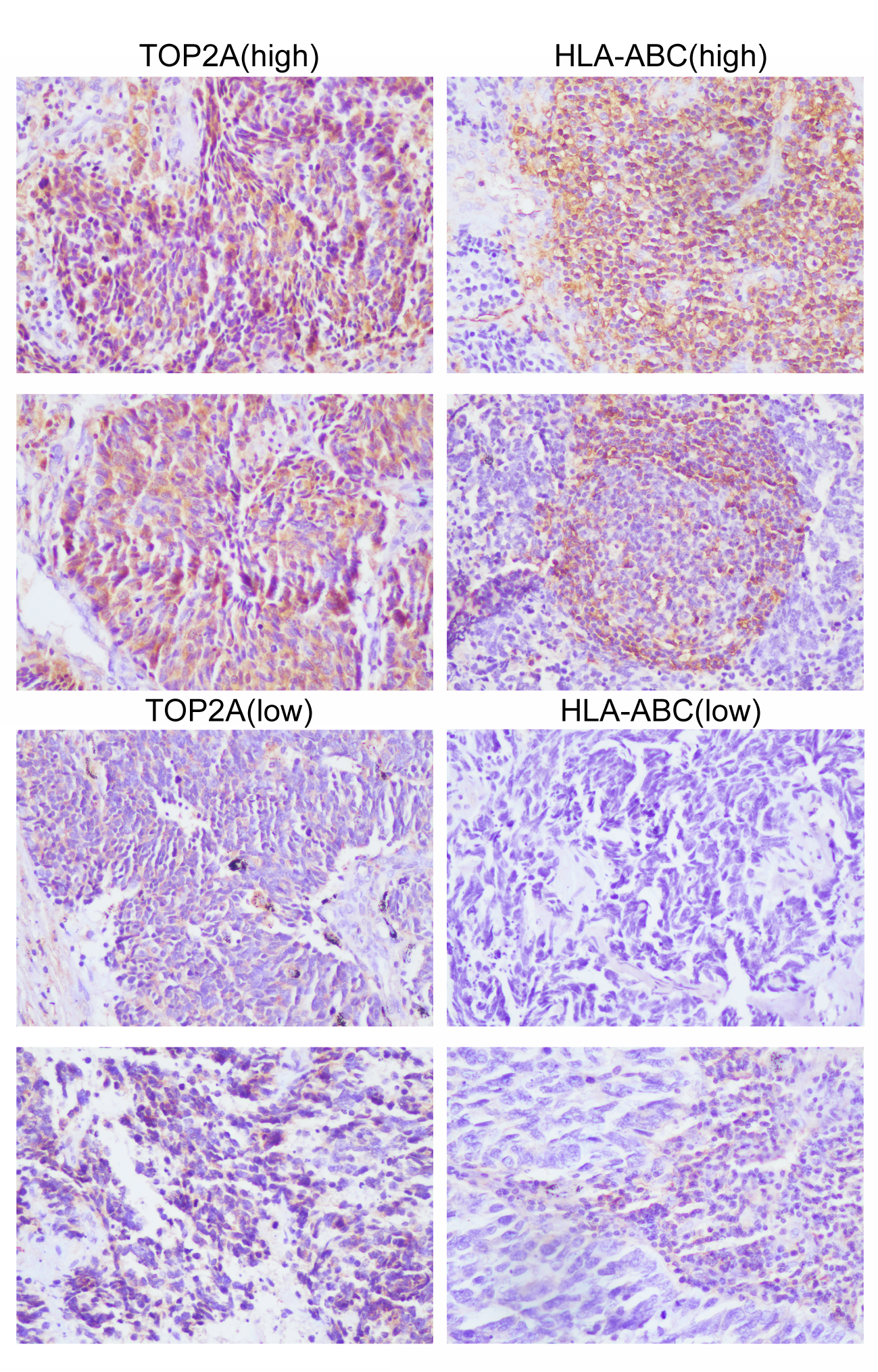

Supplement: Supplementary file 1 [file Image1.TIF]
