# Supplementary material for: Chemotherapy- and Immune-Related Gene Panel in Prognosis Prediction and Immune Microenvironment of SCLC
Source: Front Cell Dev Biol. 2022 Jun 15;10:893490. doi: 10.3389/fcell.2022.893490 (PMC9240612; doi:10.3389/fcell.2022.893490)
Supplement: Supplementary file 2 [file DataSheet1.pdf]

frontiers

Supplementary Material

Supplementary Table 1 Correlations between MSH2 expression and immune cells

Gene	Cell	Coefficient *r*	P value
MSH2	B cells naive	0.183432	0.197587
MSH2	B cells memory	-0.25879	0.066698
MSH2	Plasma cells	0.1181	0.408014
MSH2	T cells CD8	-0.24464	0.083607
MSH2	T cells CD4 naive	0.163331	0.252123
MSH2	T cells CD4 memory resting	0.097533	0.495944
MSH2	T cells CD4 memory activated	-0.16854	0.237101
MSH2	T cells follicular helper	0.135989	0.341345
MSH2	T cells regulatory (Tregs)	0.066427	0.643268
MSH2	T cells gamma delta	-0.14298	0.316871
MSH2	NK cells resting	0.115292	0.420455
MSH2	NK cells activated	0.094932	0.506431
MSH2	Monocytes	-0.04763	0.739977
MSH2	Macrophages M0	0.057513	0.688514
MSH2	Macrophages M1	0.171041	0.229389
MSH2	Macrophages M2	0.075837	0.595851
MSH2	Dendritic cells resting	-0.05111	0.721707
MSH2	Dendritic cells activated	-0.13923	0.329864
MSH2	Mast cells resting	0.173986	0.222068
MSH2	Mast cells activated	-0.09673	0.499495
MSH2	Eosinophils	-0.07686	0.591898
MSH2	Neutrophils	0.046092	0.748079

Supplementary Table 2 Correlations between BIRC5 expression and immune cells

Gene	Cell	Correlation r	pvalue
BIRC5	B cells naive	-0.13876	0.331513
BIRC5	B cells memory	-0.21863	0.12324
BIRC5	Plasma cells	-0.08172	0.567567
BIRC5	T cells CD8	-0.09651	0.500478
BIRC5	T cells CD4 naive	-0.14412	0.312997
BIRC5	T cells CD4 memory resting	-0.18506	0.193559
BIRC5	T cells CD4 memory activated	0.014846	0.917649
BIRC5	T cells follicular helper	0.230874	0.103102
BIRC5	T cells regulatory (Tregs)	0.063872	0.656114
BIRC5	T cells gamma delta	0.185218	0.193184
BIRC5	NK cells resting	0.028823	0.840873
BIRC5	NK cells activated	0.272036	0.053768
BIRC5	Monocytes	-0.37156	0.007263
BIRC5	Macrophages M0	0.071993	0.615642
BIRC5	Macrophages M1	-0.00697	0.961402
BIRC5	Macrophages M2	-0.03213	0.822523
BIRC5	Dendritic cells resting	-0.13261	0.353603
BIRC5	Dendritic cells activated	-0.19278	0.175316
BIRC5	Mast cells resting	-0.15175	0.287775
BIRC5	Mast cells activated	-0.06142	0.668557
BIRC5	Eosinophils	0.240192	0.089556
BIRC5	Neutrophils	-0.06291	0.660969

Supplementary Table 3 Correlations between CDKN2A expression and immune cells

Gene	Cell	Correlation r	pvalue
CDKN2A	B cells naive	-0.0649	0.650945
CDKN2A	B cells memory	-0.07355	0.608018
CDKN2A	Plasma cells	0.113394	0.42705
CDKN2A	T cells CD8	-0.08076	0.573184
CDKN2A	T cells CD4 naive	0.144115	0.312997
CDKN2A	T cells CD4 memory resting	-0.13161	0.357249
CDKN2A	T cells CD4 memory activated	-0.21662	0.126813
CDKN2A	T cells follicular helper	0.261566	0.063729
CDKN2A	T cells regulatory (Tregs)	0.123625	0.387426
CDKN2A	T cells gamma delta	-0.02652	0.853418
CDKN2A	NK cells resting	0.038431	0.788895
CDKN2A	NK cells activated	-0.05321	0.710003
CDKN2A	Monocytes	-0.11978	0.40246
CDKN2A	Macrophages M0	0.074481	0.603455
CDKN2A	Macrophages M1	0.253937	0.072346
CDKN2A	Macrophages M2	-0.11946	0.402616
CDKN2A	Dendritic cells resting	-0.12443	0.384326
CDKN2A	Dendritic cells activated	-0.17403	0.22194
CDKN2A	Mast cells resting	0.057695	0.687577
CDKN2A	Mast cells activated	-0.11121	0.437192
CDKN2A	Eosinophils	0.192154	0.17674
CDKN2A	Neutrophils	-0.06712	0.639818
